# Metabolomics of Prostate Cancer and Clinical Profiles Following Radiotherapy: Need for a Precision Phylometabolomics Approach †

**DOI:** 10.3390/diagnostics15243242

**Published:** 2025-12-18

**Authors:** Hakima Amri, Charles Sturgeon, David Posawatz, Mones Abu-Asab, Ryan R. Collins, Simeng Suy, Sean P. Collins

**Affiliations:** 1 Department of Biochemistry and Cellular and Molecular Biology, Georgetown University, Washington, DC 20057, USA; cs2401@georgetown.edu; 2 Boston Medical Center, Boston, MA 02118, USA; david.posawatz@bmc.org; 3 Section of Ultrastructural Biology, NEI/NIH, Bethesda, MD 20892, USA; monesasab@gmail.com; 4 Radiation Oncology, College of William & Mary, Williamsburg, VA 23185, USA; rrcollins@wm.edu; 5 Department of Radiation Oncology, Tampa General Hospital, Tampa, FL 33606, USA; ssuy@tgh.org; 6 Department of Radiation Oncology, Morsani College of Medicine, University of South Florida Health, Tampa, FL 33602, USA; collinss19@usf.edu

**Keywords:** Clinical Correlation, parsimony phylogenetics, diagnostic biomarkers, precision medicine, treatment response

## Abstract

**Introduction**: Metabolomics-based phylogenetic profiling of prostate cancer (PCa) patients before and after stereotactic body radiation therapy (SBRT) can provide insight into the way in which treatment outcomes relate to the underlying physiology and physiological responses of individual patients. It also offers the potential for helping identify precision biomarkers. **Methods**: In this study, we used integrated mass spectrometry to obtain untargeted serum metabolomics data from PCa patients (*n* = 55), which we then analyzed using a parsimony phylogenetic systems biology approach before correlating the results with the patients’ clinical parameters before and after treatment. **Results**: Radiotherapy (RT) generated five phylogenetic subgroups with distinct metabolomic profiles that did not correspond to hormonal treatment, risk assessment, metastasis, or PSA levels. PSA was neither a factor influencing clade membership nor an indicator of risk assessment or metastasis. Moreover, the hormone-treated patients did not form their own clade but were rather spread among the five clades. The same absence of correlation applied to risk assessment and metastasis. The 88 significantly altered pre-RT and 29 post-RT features showed aberrations in the metabolic pathways of purines, porphyrin, glycerophospholipids, and 2-methylglutaric acid, among others. **Discussion**: Significantly altered metabolites in a majority of patients who developed metastasis included D-tryptophan, carbamate, 5′-Benzoylphosphoadenosine, Phosphatidylcholine (PC), bilirubin, and hypoxanthine. In general, the cladogram offers a new perspective on evaluating the clinical variables that represent significant indicators of PCa progression, metastasis, and treatment response in individuals. **Conclusions**: Metabolic profiles and associated clinical phenotypes provided by this precision phylometabolomics approach may offer a deeper understanding of the metabolic factors and pathways implicated in cancer progression and metastasis and should contribute to the development of targeted treatments and more precise monitoring of cancer and cancer therapies.

## 1. Introduction

Globally, PCa ranks as one of the most prevalent malignancies among men, surpassed only by lung cancer, and it is linked to considerable incidence and mortality [1,2,3,4]. PCa treatment modalities are ideally based on tumor stage, tumor aggressiveness, and prostate-specific antigen (PSA) [5]. In combination with RT, androgen deprivation therapy (ADT) has demonstrated improvements in clinical outcomes and metastasis-free survival while acting as a radiation sensitizer for PCa patients [6,7,8]. Evidence has suggested that lowering testosterone levels below 20 ng/dL can improve patient survival and delay disease progression [9]. Mechanisms underlying the synergistic effects of ADT on RT outcomes include reductions in DNA repair protein levels [10] and the induced influx of tumor-infiltrating lymphocytes and tumor-associated macrophage infiltrates [11,12]. However, ADT has been shown to be associated with a decline in the patient’s quality of life [13]. Although disease state and risk level provide indications for different treatment options, there is a need to further subtype PCa patients through biomarker analysis to gain a better understanding of disease progression and to make possible more targeted treatment options.

The PSA is the most commonly used biomarker for PCa screening and monitoring, with >4 ng/mL generally accepted as being positive for prostate cancer. However, the accuracy and dependability of this test have been in question. The low positive predictive value (PPV) of 30% results in a substantial proportion of men who test positive having no cancer detected on biopsy and consequently being subject to overdiagnosis and overtreatment. Conversely, elevated PSA does not always accompany cancer; in one study, 15.2% of men aged 62 to 91 who had normal PSA levels were found to have PCa on biopsy [14]. These findings, along with the variability by age, race, and other factors, raise concerns as to the reliability of the PSA test for the screening and monitoring of PCa [15]. Promising studies have pointed toward the enhanced ability of serum metabolomics to make these distinctions [16].

In addition to the PSA test, which has overall reduced mortality from PCa [17], other biomarkers/diagnostic tests for PCa detection and monitoring have been developed. They include serum biomarkers, such as 4Kscore and the Beckman Coulter Prostate Health Index (PHI), and urine biomarkers, such as PCa antigen (PCA3), Exosome Dx, Select MDx, and the Mi-prostate score (MiPS). 4Kscore evaluates four kallikrein proteins—total PSA, free PSA, intact PSA, and human kallikrein 2—along with clinical parameters in order to determine the probability of having grade 2 or higher PCa. The PHI calculates its score based on total PSA, free PSA, and [−2]proPSA. These blood tests have shown promise but lack data in long-term trials and prospective trials. PCA3 is a large-chain RNA molecule that is measured in urine sediment and compared to PSA mRNA levels to obtain a PCA3 score, but it has not been able to differentiate indolent versus aggressive PCa, and its capability in monitoring progression and reclassification may be limited. Exosome Dx is a liquid biopsy that combines a three-gene signature based on exosomal DNA to provide an IntelliScore on a scale from 0 to 100, with 15.6 being the cut-off for increased likelihood of grade 2 or higher PCa. While it has demonstrated a sensitivity of 92% and an NPV of 91% with this cutoff, prospective validation is still underway [18]. SelectMDx measures DLX1 and HOX6 mRNA levels in post-digital rectal exam (DRE) urine [19]. MyProstateScore (MPS, previously MiPS) measures TMPRSS2:ERG and PCA3 in post-DRE urine along with clinical information [20]. In general, these tests have shown early promise, but they lack data in long-term, prospective, and surveillance trials [21]. Their reliance on single or a few biomarkers may be a barrier to understanding the dynamic and complex features of disease [22].

Radiation metabolomics has emerged as a useful tool in the characterization of dysregulated metabolic pathways involving serum amino acids, fatty acids, and lipids/sphingolipids, all of which have been found to be radiation-responsive. Analyses of these dysregulated molecules have been recommended for monitoring patient clinical parameters during treatment [23,24,25,26,27,28]. Changes in radiation-responsive biomarkers such as p53, MDM2, and Bax have also been associated with clinical RT outcomes. However, to date, few studies have focused on predictive biomarkers specific to RT, focusing rather on human cell culture or animal studies (nonhuman primates, rats, and mice) to identify disease-related biomarkers [24,25,26,29,30]. The metabolomics-based investigation of responses to PCa RT provides valuable insight into PCa occurrence, progression, and response to treatment.

The application of multi-omics analyses has grown significantly in recent years for both molecular-level disease profiling and comprehensive therapy monitoring [16,25,31]. Relative to other “omics” such as genomics, transcriptomics, and proteomics, metabolomics provides a unique profile of metabolites that reflect systemic or cellular physiology at specific stages of disease and response to treatment [32,33,34]. Unfortunately, the heterogeneity of cancer complicates the discovery of predictive and therapeutic biomarkers from omics data. We have demonstrated that parsimony phylogenetic analysis, as a robust systems biology synthesis, offers an analytical paradigm that can manage the heterogeneity and high dimensionality of omics data, produce meaningful biological classifications, and realize differential disease profiles [35,36,37]. Researchers have applied this method to analyze mass spectrometry (MS) proteomic data in cancer, gene expression across various disease states, and systems biology [35,38,39,40,41]. Our earlier report on applying parsimony phylogenetic analysis to metabolomics data from PCa patients demonstrated its utility in stratifying cancer patients and identifying subtypes based on treatment or disease status [37]. Here, we utilize precision phylometabolomics to analyze the correlation of PCa patient serum metabolomic profiles with their clinical parameters before and after treatment. We ought to provide an in-depth analysis of our matched pre- and post-radiation human serum samples of the association of metabolic profiles with clinical factors, including the following: (a) known PCa prognostic variables (such as the Gleason score at diagnosis), (b) impact of the timing of serum collection relative to RT on metabolic profile, and (c) profile differences based on clinical outcomes using parsimony phylogenetic analyses. We hypothesize that the present study may describe the metabolomic profile changes induced in response to RT and that changes in metabolite patterns after RT might yield valuable insight into the causes of the diverse treatment outcomes observed clinically.

## 2. Materials and Methods

### 2.1. Mass Spectrometry-Based Global (Untargeted) Metabolomics

Frozen human serum samples (Lomabrdi Cancer Center blood bank, Washington, DC, USA), collected from whole blood before and after RT, were stored at −80°C. In brief, thawed serum samples were mixed with 40% ACN, 25% methanol, and 35% water containing internal standards (debrisquinone for positive mode and nitrobenzoic acid for negative mode) [37]. Following centrifugation, the supernatant was speed-vacuum-dried at room temperature and reconstituted in a solution of 5% methanol, 1% ACN, and 94% water. The specimens were centrifuged again at 13,000 rpm for 20 min at 4 °C, and an aliquot of the supernatant solution was injected into a reverse-phase 50 × 2.1 mm Acquity 1.7-μm C18 column (Waters Corp., Milford, MA, USA) coupled to a UPLC-ESI-QTOF-MS system (Xevo G2 Q-TOF, Waters Corporation, Milford, MA, USA). The mobile phase consisted of a gradient from 2% ACN in water containing 0.1% formic acid (A) to 98% ACN in water containing 0.1% formic acid (B). Mass spectrometry analysis was performed in both negative-ion (ESI-) or positive-ion (ESI+) electrospray ionization modes with the following system parameters: resolving time, 10 min; flow rate, 0.5 mL/min; capillary voltage, 3200 V; cone voltage, 20 V in negative mode and 35 V in positive mode; desolvation gas flow, 800 L/h; temperature, 350 °C; cone gas flow, 25 L/h; source temperature, 120 °C. For accurate mass maintenance, the LockSpray interface of sulfadimethoxine (311.0814 [M+H]^+^ or 309.0658 [M-H]^−^) at a concentration of 250 pg/μL in 50% aqueous ACN was used at a rate of 150 μL/min [42]. To control for the accuracy and robustness of the method, eight quality control (QC) injections of 80 µL each from the sample pool were injected every ten samples. In every 50th sample, we doubled that sample volume to ensure accuracy. The cycle of injections was repeated on three consecutive days in the same batch/machine columns. All pre- and post-RT samples were run in the same batch. Integrated MS data within the mass range of 50 to 850 mass-to-charge ratio (m/z) were processed to generate a multivariate data matrix using XCMS 3.8 [43].

### 2.2. Phylogenetic Modeling of MS Data: MS-Based Phylometabolomics

Parsimony phylogenetic analysis employs two algorithms: a new parsing algorithm of m/z values (OmicsTract) developed and patented by the authors and a phylogenetic algorithm (MIX). Feature data values extracted from XCMS files were polarized by comparing them to an outgroup of healthy individuals and then coded as derived (abnormal) or ancestral (normal) states using the OmicsTract program [35]. The parsing algorithm identifies novel or vanished MS peaks and peaks signifying up- or downregulated metabolites and scores them as derived or ancestral. The phylogenetic algorithm uses the latter scores to produce a biologically meaningful classification of the specimens rather than converting the continuous values into discontinuous ones through the assessment of each peak’s values against those of the normal range, producing a matrix of polarized values (0s and 1s). We used all expression data points of all specimens in the analysis without any a priori selection of any specific group or groups of data. Further details about the development and application of the analytical method, OmicsTract, could be found in our previously published work [35,44]. To generate a hierarchical classification of specimens on a cladogram, the phylogenetic analysis was conducted with MIX, the maximum parsimony program of PHYLIP ver. 3.57c [45]. MIX was run with randomized and nonrandomized inputs, and no significant differences were observed between the two options. Phylogenetic trees were drawn using TreeView [46].

### 2.3. Data Collection and Metabolic Pathway Analysis

The pre-processing of the raw data, including peak detection and alignment, was conducted using the XCMS (3.8) software. The resultant three-dimensional data matrix consisting of m/z ratios with retention times and feature intensities was subjected to multivariate data analysis. Debrisoquine and 4-nitrobenzoic acid were identified as internal standards for the positive and negative modes, respectively. The former showed m/z 176.117 and a retention time of 1.705 min, and the latter showed m/z 166.014 with a retention time of 4.063 min. The analysis’s reproducibility was attested to by obtaining standard runs for both positive and negative ion modes, all of which fell within the ppm error range. All feature data, including m/z values, retention times, and normalized intensities, were recorded in a CSV file and subjected to parsimony phylogenetic analysis for stratification and inter-group comparisons. The analysis was performed using the ChemSpider and METLIN database (Metabolite and Tandem MS Database; http://metlin.scripps.edu/, accessed on 5 December 2025) at an accuracy of 5 ppm with either hydrogen, sodium, or potassium adducts. For the presumptive identification of altered features, we queried each molecular mass and its likely chemical formula against databases, including HMDB 3.0 [47], MMCD [48], METLIN 3.7.1 [49], and LipidMaps [50]. Pathway enrichment analysis was conducted using the Kyoto Encyclopedia of Genes and Genomes (KEGG 12.0) [51] and ConsensusPathDB [52] databases to identify pathways frequently observed and potentially linked to RT and disease outcomes.

## 3. Results

### 3.1. Patient Characteristics and Treatment Planning

This study compared patients with clinically diagnosed and histologically confirmed localized prostate adenocarcinoma before and after RT. Samples were collected before prostate membrane-specific antigen (PMSA) PET/CT scans were available. Treatment planning and fiducial tracking delivery were performed as previously described [37,53,54,55]. Between March 2008 and September 2012, patients with PCa were treated with SBRT with a prescription dose of 19.5 Gy delivered in three doses of 6.5 Gy over 3 to 5 days, followed by intensity-modulated radiation therapy (IMRT) with a prescription dose of 45–50.4 Gy in 25–28 doses of 1.8 Gy daily. Blood samples were obtained from all patients prior to RT and one day after patients had completed their prescribed radiation fractions. This study was conducted with the informed consent of all participating patients and received approval from the Institutional Review Board of Georgetown University (IRB 2009–096). Patient characteristics are further summarized in Table 1. Patient age ranged from 52 to 90 years, with a median age of 68. The pre-treatment PSA levels in this cohort ranged from 1.9 to 26.6 ng/mL, with a median level of 8.1 ng/mL. Patients were categorized into three risk groups based on the D’Amico classification [56]: 14 patients were low risk, 29 were intermediate risk, and 7 were high risk. ADT was prescribed to 23.6% of the intermediate or high-risk patients undergoing radiation therapy.

**Figure 1:**
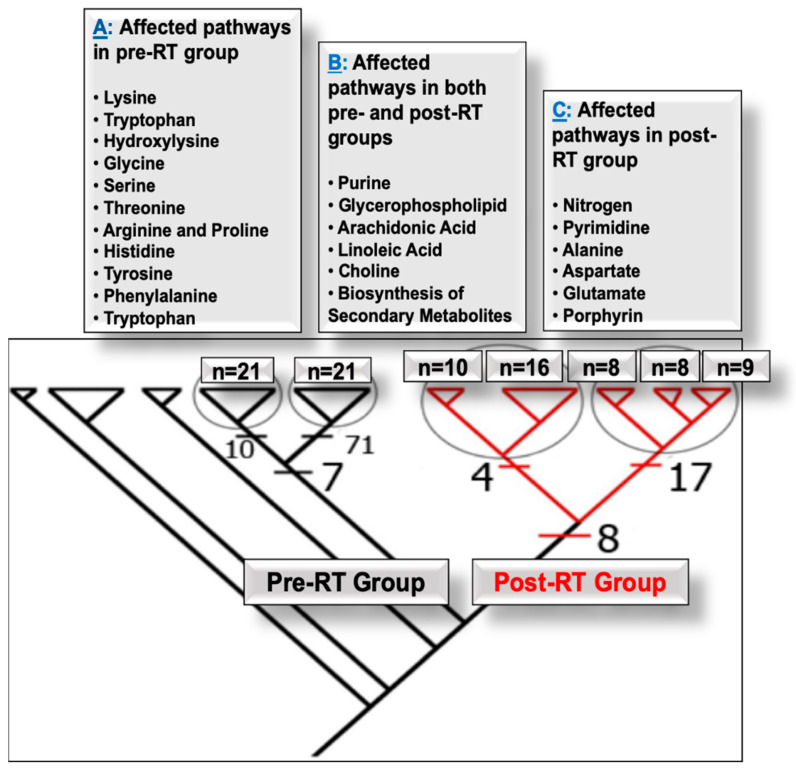
Parsimony phylogenetic analysis of metabolomics data of PCa patients. The clades in black color represent patients before RT and are delineated by 88 altered serum metabolites or synapomorphies; among those, 7 synapomorphies are shared among the two circled clades. Those clades are delineated with an additional 71 (right) and 10 (left) synapomorphies. The clades in red color represent patients after RT and are delineated by 29 altered serum metabolites or synapomorphies; among those, 8 synapomorphies are shared among the two circled clades. Those clades are delineated with an additional 17 (right) and 4 (left) synapomorphies. Panels (A, B, C) represent the identified metabolites and pathways.

Parsimony phylogenetic analysis discriminated between serum samples of pre- and post-RT (Figure 1). Pre-RT samples formed four clades, depicted in black, while post-RT samples clustered into five subclades, depicted in red, within the right main clade. The topography of the subclades shows an abundance of metabolites that could be attributed to intra-group variations, including factors like additional hormone therapy, risk assessments, PSA levels, specific demographic features, and, critically, patients’ response to treatment. The number of synapomorphies that delineate the subclades is the shared derived characters among the patients in that group. Common synapomorphies for each clade were subsequently identified by examining the nodes of the cladogram. We defined the number of synapomorphies per clade as the number of significant metabolites, with these counts annotated directly on the relevant clades (Figure 1). By extracting the synapomorphies from each node, we were able to identify the corresponding metabolites.

Of the 109 compounds significantly altered between the two groups, 26 were putatively identified by examining changes in peak intensity and performing database searches. Eleven compounds were putatively identified in the pre-RT group: D-tryptophan, dihydrosanguinarine, methylglutaric acid, hypoxanthine, tetrahydroisoquinoline, dabigatran etexilate mesilate, and unknown compounds: m/z 158.0015368, m/z 339.1655, m/z 146.1412, PE(22:6(4Z,7Z,10Z,13Z,16Z,19Z)/18:0), and PC(22:6(4Z,7Z,10Z,13Z,16Z,19Z)/18:0). Fifteen distinct compounds were putatively identified in the post-RT group through database searches, including bilirubin, carbamic acid, phosphoric acid, phthalic acid, 5’-benzoylphosphoadenosine, 8-(Methylsulfonyl)-2,2-bis(trifluoromethyl)-2,3-dihydro-4H-[1,3,5]triazino [2,1-b][1,3]benzothiazol-4-one, m/z 221.992939089, m/z 343.032024687, m/z 632.2994, m/z 595.3515, m/z 262.2532, m/z 723.5443, PC(18:3(9Z,12Z,15Z)/20:5(5Z,8Z,11Z,14Z,17Z)) or PC(38:8), PC(18:1(11Z)/16:1(9Z)) or PC(34:2), and PE(18:3(9Z,12Z,15Z)/20:3(5Z,8Z,11Z)) or PE(38:6).

Clinical data correlations were made based on the total of 88 significantly altered features (synapomorphies) listed at the nodes of pre-RT clades and the 29 significantly altered features listed at the nodes of post-RT clades. Predominant aberrations in the metabolic pathways of nitrogen, pyrimidine, purine, porphyrin, alanine, aspartate, glutamate, and glycerophospholipid defined the radiation-responsive serum metabolome. Bilirubin, phthalic acid, 5’-benzoylphosphoadenosine, and carbamic acid were altered in all of the metastatic patients in the post-RT group. Moreover, all of the pre-RT patients in the high-risk group carried hypoxanthine, while all of the post-RT patients with high risk carried phthalic acid. In addition, D-tryptophan, hypoxanthine, tetrahydroisoquinoline, dihydrosanguinarine, and methylglutaric acid were observed in pre-RT metastatic patients.

All of the seven patients in the high-risk group carried the following compounds pre-RT: hypoxanthine, m/z 159.0093618, m/z 120.0038, m/z 380.772, and m/z 197.08. In the post-RT group, 100% of the patients who were high risk and who were, in addition, metastatic carried the following compounds: phthalic acid, 5′-Benzoylphosphoadenosine, bilirubin, PEPC, m/z 416.91035669, m/z 632.2994, m/z 312.0288, m/z 262.2532, m/z 184.0735, m/z 723.5443, and m/z 701.561. Metastasis occurred in eleven patients after treatment, six of whom had been classified as intermediate risk, three of whom were low risk, and two of whom were at high risk. Only four of these patients had received additional hormone therapy (ADT). One of the patients who had received ADT was in the treatment category of SBRT/IMRT with intermediate risk. Seven out of eleven patients carry D-tryptophan pre-RT; six out of seven high-risk patients carry carbamic acid; five out of seven carry phosphoric acid post-RT (Table 2).

### 3.2. Distribution of Putatively Identified Compounds Before RT

m/z 203.0233 (n = 31), which was putatively identified as D-tryptophan, was uniquely observed in 31 of the 35 patients in the pre-RT group. D-tryptophan was significantly altered in seven metastatic patients in the pre-RT group. m/z 333.100108 (n = 32), which was putatively identified as Dihydrosanguinarine, was observed in 32 of the 55 patients in the pre-RT group. Dihydrosanguinarine was significantly altered in four metastatic patients in the pre-RT group. None of those patients received additional ADT. m/z 792.5539 (n = 20)-792.0765, which was putatively identified as PE, was observed in 20 patients in the pre-RT group out of 55. PE was significantly altered in four metastatic patients in the pre-RT group, one of whom received additional ADT. m/z 146.1412 (n = 20), which was putatively identified as methylglutaric acid, was observed in 20 patients in the pre-RT group out of 55. Methylglutaric acid was significantly altered in three metastatic patients in the pre-RT group, one of whom received additional ADT. m/z 137.046 (n = 52), which was putatively identified as hypoxanthine, was observed in 52 patients in the pre-RT group out of 55. Hypoxanthine was significantly altered in all of the (n = 8) metastatic patients in the pre-RT group, two of whom received additional ADT. m/z 133.0861 (n = 2), which was putatively identified as tetrahydroisoquinoline, was observed in 2 patients in the pre-RT group out of 55. Tetrahydroisoquinoline was significantly altered in one metastatic patient in the pre-RT group, with the high-risk group not receiving ADT. m/z 834.6015 (n = 20), which was putatively identified as PC, was observed in 20 patients in the pre-RT group out of 55. PC was significantly altered in four metastatic patients in the pre-RT group, none of whom received ADT. m/z 724.3153 (n = 32), which was putatively identified as dabigatran etexilate mesilate and usually prescribed as an anticoagulant drug; m/z 158.0015368 (n = 11); m/z 339.1655 (n = 11); and m/z 146.1412 (n = 20) were not present in any of the low-risk but metastatic patients.

### 3.3. Distribution of Putatively Identified Compounds After RT

m/z 61.0163783457 (n = 49), which was putatively identified as carbamic acid, was observed in 49 patients in the post-RT group out of 55. Carbamic acid was significantly altered in all of the metastatic patients in the post-RT group. m/z 586.09720 (n = 55), which was putatively identified as phthalic acid, was observed in all of the patients (100%) in the post-RT group. Phthalic acid was significantly altered in all of the metastatic patients in the post-RT group. m/z 451.08929 (n = 55), which was putatively identified as 5′-Benzoylphosphoadenosine, was observed in all of the patients (100%) in the post-RT group. 5′-Benzoylphosphoadenosine was significantly altered in all of the metastatic patients in the post-RT group. m/z 358.092987485 (n = 40), which was putatively identified as phosphoric acid, was observed in 40 of the patients in the post-RT group. Phosphoric acid was significantly altered in 5 out of 11 (45.5%) metastatic patients in the post-RT group. m/z 585.2715 (n = 55), which was putatively identified as bilirubin, was observed in all of the patients (100%) in the post-RT group. Bilirubin was significantly altered in all of the metastatic patients in the post-RT group. m/z 758.5717 (n = 55), which was putatively identified as PC, was observed in all of the patients (100%) in the post-RT group. PC was significantly altered in all of the metastatic patients in the post-RT group. m/z 802.5395 (n = 47), which was putatively identified as PC, was observed in 47 patients in the post-RT group out of 55. m/z 764.526 (n = 51), which was putatively identified as PE, was observed in 51 patients in the post-RT group out of 55.

Post-RT compounds m/z 632.2994 (n = 54), bilirubin, PC, m/z 312.0288, m/z 262.2532, m/z 723.5443, and m/z 701.5618 were significantly altered in all metastatic patients. Post-RT significantly altered unidentified metabolites’ m/z values, and they are as follows: 343.032024687 (n = 8), 449.106011787 (n = 14), 221,992939089 (n = 3), 416.91035669 (n = 52) and 419.992671642 (n = 3), 132.0795 (n = 17), 139.0741 (n = 19), 632.2994 (n = 54), 763.5739 (n = 28), 593.3355 (n = 31), 595.3515 (n = 35), 449.106011787 (n = 14), 723.4896 (n = 35), 312.0288 (n = 54), 262.2532 (n = 53), 184.0735 (n = 49), 723.5443 (n = 54), 701.5618 (n = 55), and 838.5146 (n = 22). Among those, m/z 416.91035669 was seen in 7 out of 11 metastatic patients (63.6%). m/z 419.992671642 (n = 3), m/z 221.9929391 (n = 3), and m/z 343.032024687 (n = 8) were not present in any of the low-risk but metastatic patients.

## 4. Discussion

Since cancer progression is the accumulation of traits or features that have resulted from evolution over time, phylo-oncology or cancer phylogenetics refers to diverse applications of phylogenetic algorithms to the analysis of cancer data [36]. Edison and colleagues have previously reported that “the grand challenge is to identify and map all metabolites onto metabolic pathways, to develop quantitative metabolic models for many model organisms and to relate organism metabolic pathways within the context of evolutionary metabolomics, i.e., phylometabolomics” [57]. The authors define “phylometabolomics” as the comparative analysis of the evolution of metabolism and metabolic networks in a phylogenetic context. A *phylometabolomics* approach enables us to identify the evolution of metabolic networks and also how these respond to internal and external stressors, such as toxicity, to reveal the association between the phenotype(s) induced by these stressors [58]. In this concept, since cancer is an evolutionary condition that involves genetic modifications and clonal production, a *phylometabolomics* approach as an evolutionary method of analysis is suggested to reveal altered *metabolites and metabolic networks during disease occurrence and the treatment period.* The multiphasic nature of cancer progression, combined with possible multiple developmental pathways [59,60,61,62], entails the presence of significant metabolomic changes for each metabotype. Thus, the metabolite profile is suggested as a hierarchical and continuous accumulation of metabolic change over time rather than one or a few simple, distinct metabolic patterns. Phylometabolomics is an evolutionary analytical tool that sorts mass-to-charge (m/z) values into derived (apomorphic) or ancestral (plesiomorphic) and then classifies specimens according to the distribution pattern of their apomorphies into clades (a group composed of all specimens sharing the same apomorphies) sharing unique metabolite changes (synapomorphies) among its specimens [44,63,64,65,66,67].

Precision radiation oncology is an emerging field that demonstrates the potential to facilitate the development of a personalized treatment plan for individuals based on the ability of ionizing radiation to alter metabolic processes and metabolite levels governing a complex network of molecular and cellular responses [68]. In addition, the system-level analysis of ionizing radiation-responsive metabolites and pathways allows for the determination of novel disease and treatment-related biomarkers [69]. Challenges in radiobiology involve the accurate prediction of tumor radioresistance and determining normal tissue radiosensitivity [70]. Lastly, radiation-induced toxicities are suggested to derive from radiation damage, and a radiogenomic modeling study indicated that the differential genetic backgrounds of individuals lead to radiation sensitivity variations and predictive radiation toxicities [71].

Medipally and colleagues [5] investigated the potential of Fourier transform infrared (FTIR) spectroscopy for monitoring radiotherapeutic response, radiation-induced proteomic changes, and mechanisms of resistance of human tumors. Patient-specific radiation-induced expression differences have been obtained with proteins and pathways for which their abundance is consistently perturbed following radiation. Using non-invasive blood serum samples, these expression differences are appropriate for monitoring radiotherapeutic response in PCa patients and could lead to individualized patient RT.

**Figure 2:**
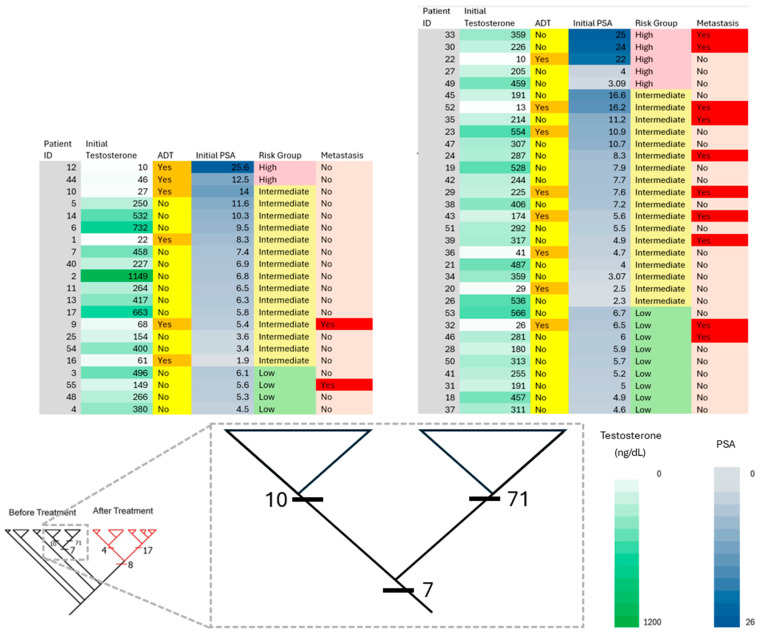
Phylogenetic classification of patients and their clinical profiles before RT. The clinical correlates (PSA values, testosterone levels, ADT, risk assessment, and metastasis involvement) are color-coded following a color intensity gradient from low to high values. The two major clades before RT (circled in Figure 1) are amplified, and the patients grouped in the respective clades are listed above it.

**Figure 3:**
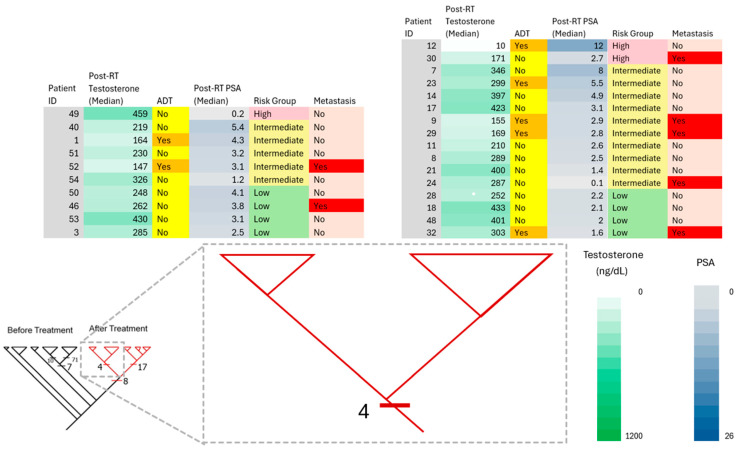
Phylogenetic classification of patients and their clinical correlates after RT. The clinical correlates (PSA values, testosterone levels, ADT, risk assessment, and metastasis involvement) are color-coded following a color intensity gradient from low to high values. The two major clades after RT (circled in Figure 1, left clade) are amplified, and the patients grouped in the respective clades are listed above it.

**Figure 4:**
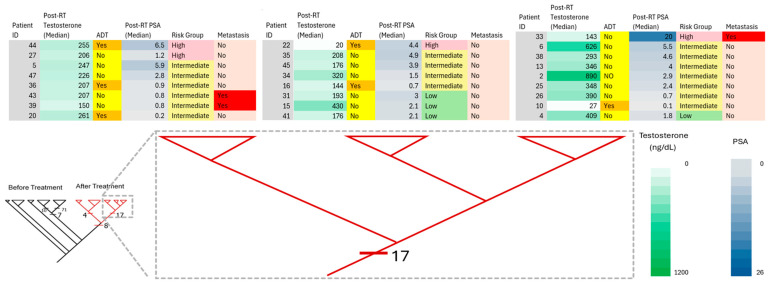
Phylogenetic classification of patients and their clinical correlates after RT. The clinical correlates (PSA values, testosterone levels, ADT, risk assessment, and metastasis involvement) are color-coded following a color intensity gradient from low to high values. The two major clades after RT (circled in Figure 1, right clade) are amplified, and the patients grouped in the respective clades are listed above it.

Pre-treatment assessments of prostate-specific antigen (PSA), tumor stage (TNM), and the pathologic Gleason score have long been used to predict an individual PCa patient’s response to RT [72]. Pre- and post-treatment PSA values have been applied to predict disease-free survival and long-term response to RT. Relapse is defined as a rise in PSA levels at 2× above its PSA nadir (Phoenix definition). However, commenting on post-RT PSA values with respect to monitoring tumor treatment response can sometimes be misunderstood as recurrence due to PSA elevations following therapy. A further common clinical implication after RT is the decline in the levels of PSA measurements, used routinely to monitor treatment outcomes. Even though PSA is used as a tool for PCa screening, prognosis, and monitoring therapeutic response, elevations in PSA levels have also been obtained in cases such as inflammation and non-malignant benign prostate hyperplasia. Furthermore, these diseases are characterized by heterogeneity at the cellular/tissue level. In the present study, we found that patients who developed metastasis in the intermediate- and low-risk groups, pre-RT PSA levels were above 4 ng/mL, and post-RT PSA levels were in the low range: <4.0 ng/mL (Figure 2, Figure 3 and Figure 4). Furthermore, 12.5% of patients with PSA levels falling in the normal range (<4.0 ng/mL) in the post-RT intermediate-risk group and 28.6% of patients with PSA levels falling in the normal range (<4.0 ng/mL) in the post-RT low-risk group developed metastasis (Figure 3 and Figure 4). Our data suggests that the omics-based profiling of multiple biomarkers, rather than one single gene/biomarker, may provide better reliability for future screening/diagnosis/monitoring/treatment strategies.

As a clinical tool, phylometabolomic classification can be used to perform early detection, diagnosis, grading, prognosis, and post-treatment evaluation through a parsimony analysis where the location of a specimen within the cladogram (i.e., the classification) will indicate its pathologic status. When applied, our parsimony phylogenetics algorithm revealed the correlation of PCa metabolome data with the clinical variables in this study. While evolution-based analytical methods like parsimony phylogenetics are established as effective tools for high-dimensional and heterogeneous data, their application to the analysis of RT PCa is novel [37,39,41,73,74,75]. The analysis produced only one most parsimonious cladogram, a strong sign that the dataset was free of experimental artifacts and batch issues [37]. Our findings indicate that applying this systems biology strategy to metabolomics data enabled the stratification of cancer patients and the identification of subtypes based on their treatment, response to treatment, or disease status. This precision phylometabolomics approach has allowed the identification of altered compounds and pathways pre- and post-RT that may potentially be implicated in PCa progression, metastasis, and treatment response.

### 4.1. Pathways Altered in PCa Patients Pre- and Post-RT

#### 4.1.1. Pre-RT Alterations

Our previous study [37] provided novel information on the pre-RT alteration of methylglutaric acid, which is analyzed further in this paper. 2-methylglutaric acid has been suggested to have critical importance for tumor progression in both the cell [76] and tissue studies [77] of PCa. Due to its alteration only in the pre-RT group, our findings suggest that not only might 2-methylglutaric acid be a relatively unexplored compound implicated in PCa progression, but it may also be a particularly radio-sensitive metabolic pathway.

#### 4.1.2. Pre- and Post-RT Alterations

We have previously reported predominant alterations in amino acid metabolic networks in pre-RT groups and purine, glycerophospholipid, arachidonic acid, and linoleic acid in both pre- and post-RT groups [37]. Significant alterations in glutathione (GSH), pyrimidine, and purine metabolism have also been reported upon radiation exposure in other studies [78]. Uncontrolled purine metabolism has been linked to cancer progression [79], and its dysregulation could be an early marker used in clinical detection and monitoring of PCa.

This study found that serum glycerophospholipids PE (22:6/18:0) and PC (22:6/18:0) were affected pre-RT, while PC (18:3/20:5), PC (18:1/16:1), and PE (18:3/20:3) were impacted post-RT. These findings are consistent with prior research suggesting altered fatty acid biosynthesis and lipid-based signaling play a role in tumorigenesis and the RT-based metabolome [80]. Prior research by Guo and colleagues has also shown a weak but noteworthy negative correlation between PE levels and prostate cancer risk [81]. Furthermore, PC alterations have been seen with radiation toxicity in head and neck cancer patients [82], while certain PCs (LPC 18:1, LPC 20:4, LPC 20:3, and LPC 22:6) were associated with lung cancer progression. These findings align with the observations in this study, where alterations in PC metabolism were seen in pre- and post-RT groups.

Metabolomic pathways altered in both pre- and post-RT—purine, glycerophospholipid, arachidonic acid, and linoleic acid—may be involved in PCa cancer progression but may have a less pronounced role in the response to RT or may be more radio-resistant than other analyzed pathways.

#### 4.1.3. Post-RT Alterations

Nitrogen, pyrimidine, alanine, aspartate, glutamate, and porphyrin pathways were only affected in the post-RT group, suggesting their role in treatment response to RT. Nitrogen, by way of its involvement in amino acid metabolism [83], particularly glutamine, is implicated in cancer progression [84]. The nitrogen pathway was one of the pathways predominantly affected in the post-RT group, suggesting its radiosensitivity and potential involvement in the therapeutic mechanism of RT. Whether RT contributes to cancer progression through the alteration of the nitrogen pathway or whether it ameliorates the nitrogen pathway already altered through PCa cancer progression remains unknown and should be the subject of further investigation.

Altered porphyrin metabolism has been demonstrated in other types of cancer [82] and was demonstrated here by predominant post-RT alterations in bilirubin and phosphoric acid, byproducts of porphyrin metabolism. Furthermore, the association between cancer and the biosynthesis of porphyrins through abnormal heme synthesis and subsequent release of reactive oxygen species (ROS) has been well documented, with porphyrins now being used for theranostic purposes [85,86] and in conjunction with RT for improved tumor response in mouse models [87].

Compounds that were predominantly altered post-RT may be implicated in the RT treatment response, and they could be used in the future as unique profiles of PCa response to RT. This could also indicate compounds that will need to be supplemented or replenished after being altered, dysregulated, or depleted by RT to reduce its negative effects.

### 4.2. Compounds Associated with PCa Progression and Metastasis

In this study, an altered RT serum metabolome of PCa patients that represented a unique group distinct from the pre-RT cancer based on metabolic response to RT has been revealed in our previous work [37]. We previously identified alterations in the metabolic pathways of nucleotides, porphyrin, heme, glycerophospholipids, and amino acids. Here, we focused on longer-term outcomes of PCa patients treated with RT and analyzed the compounds and associated metabolic pathways that are implicated with longer-term outcomes such as metastasis. Notably, the precision biosignatures of D-tryptophan and hypoxanthine were significantly altered pre-RT in a majority of patients who developed metastasis, while carbamic acid, phosphoric acid, 5′-Benzoylphosphoadenosine, PC, and bilirubin were significantly altered in a majority of patients post-RT who developed metastasis. In total, 7 out of 11 (63.6%) patients who developed metastasis had significant alterations in D-tryptophan, and 100% metastatic patients had significant alterations in hypoxanthine biosignature pre-RT. Moreover, six out of seven (85.7%) metastatic patients had significant alterations in carbamic acid post-RT, and five out of seven (71.4%) had significant alterations in phosphoric acid post-RT (Table 2). PC and bilirubin were significantly altered in all (100%) metastatic patients post-RT.

D-tryptophan was altered pre-RT in 63.6% of patients who developed metastasis (Table 2). Previous studies have elucidated the role of tryptophan catabolism in immunosuppressive cancer progression, suggesting that tryptophan provides an essential immune-regulatory function in the body’s ability to defend against cancer. Tryptophan catabolism has been found to be mediated primarily by the enzymes indoleamine 2,3-dioxygenase 1 (IDO1) and tryptophan 2,3-dioxygenase (TDO) through the kynurenine pathway [88]. Increased kynurenine pathway activity is associated with the reduced function of natural killer cells [89] and T-cells [90], the activation of immunosuppressive T-regulatory cells [91], and the increased expression of the checkpoint molecule PD-1 [92]—all of which contribute to tumor progression. In addition, a risk model published by Shao and colleagues validates tryptophan and its molecular subtypes as dependable indicators for the biochemical recurrence of prostate cancer following radical prostatectomy in PCa patients [93]. Our results demonstrating that D-tryptophan is altered in the majority of patients who developed metastasis provide further support for tryptophan’s involvement in the tumoricidal immune defense mechanism. Furthermore, it suggests that, beyond tumor progression, the dysregulation of tryptophan may be a potential mechanism involved in metastasis.

Bilirubin, a strong antioxidant and product of heme catabolism, was found to be significantly altered post-RT in all of the patients who developed metastasis in this study (Table 2). A recent *Nature* study demonstrated a U-shaped association between cancer risk and bilirubin levels, indicating that bilirubin levels on both the high and low ends of the normal range may increase PCa risks. Total death, however, was associated with low bilirubin levels only [94]. These findings suggest a role for dysregulated bilirubin metabolism in PCa progression and the development of metastasis, which should be further explored.

PC was altered post-RT in 100% of PCa patients who developed metastasis. Abnormal PC metabolism and glycerophospholipid metabolism have been observed in multiple types of cancers, wherein PC precursors and breakdown products interact with oncogenic signaling pathways [95]. PCa patients, in particular, were found to have decreased levels of serum PC compared to benign prostatic hyperplasia control, suggesting abnormal choline metabolism in PCa tissue [96] as an etiological mechanism that differentiates these prostate pathologies. Giskeødegård and colleagues [96] provide a potential basis for the observed alterations in PC seen in metastatic patients in our study, and they provide further support for phylometabolomic analysis as an enhanced method to distinguish PCa from BPH than PSA.

Hypoxanthine, a naturally occurring purine derivative, was altered in 100% of PCa patients who developed metastasis in this study. Due to its role in cell proliferation, impaired purine metabolism is associated with cancer progression [79], which is concordant with our observations in which numerous purine metabolites were altered in both pre- and post-RT groups. Hypoxanthine has been reported as a diagnostic biomarker for several tumors in cells and tissues, and it was found to be altered by radiation exposure in non-human primates [97,98] and elevated in non-Hodgkin lymphoma [97] and colorectal cancer [98]. A similar analysis could be conducted with PCa to better ascertain the effect of dysregulated hypoxanthine on cancer progression, especially in relation to other purine metabolites.

It is also noteworthy to draw attention to the unidentified m/z values, such as predominantly altered pre-RT compounds m/z 312.0288, m/z 262.2532, m/z 723.5443, and m/z 701.5618 and post-RT compound m/z 416.91035669. All altered pre-RT compounds were altered in 100% of metastatic patients, while m/z 416.91035669 was altered post-RT in 7 out of 11 metastatic patients (63.6%). Further research to identify these compounds and their role in tumor progression, metastasis, and treatment response is necessary and could yield novel targets for precision medicine applications. Interestingly, m/z 419.992671642 (n = 3), m/z 221.9929391 (n = 3), and m/z 343.032024687 (n = 8) were not present in any of the pre-RT low-risk patients that developed metastasis.

Amongst the compounds that were identified as being altered in all or most of the patients who developed metastasis, carbamic acid, 5′-Benzoylphosphoadenosine, phthalic acid, and phosphoric acid, to our knowledge, have few documented associations with cancer progression and metastasis, let alone with PCa in particular. Thus, further investigation should help elucidate the role of these compounds in the progression, metastasis, and RT response of PCa and other types of cancer.

The observed alterations in D-tryptophan, bilirubin, PC, and hypoxanthine pathways shed further light on the metabolic mechanisms by which PCa progresses into metastasis. These results could provide an additional basis for further investigation into the specific mechanisms by which each metabolic pathway influences PCa progression, development of metastasis, and response to RT. These findings also provide support for a precision phylometabolomics approach to the monitoring of cancer progression and metastasis, whereby the observed dysregulation of the aforementioned compounds and their respective pathways may prompt moving a patient into a high-risk category and adopting a treatment plan accordingly. This could enable potential targeted treatment options in the scope of precision radiation oncology and beyond.

## 5. Conclusions

While the main focus has been on identifying driver gene mutations and genomic instability causing urological pathologies, including cancer, such as LoY [99], recent years have seen an increase in the number of investigations of various metabolomic markers in disease progression and treatment response. However, besides our first paper on this cohort, there have been no studies investigating PCa treatment response in humans using phylometabolomics [37]. While this metabolomic profiling enabled by parsimony phylogenetics allowed us to identify metabolomic markers for use in prognosis, treatment selection, and therapeutic management, larger-scale human PCa studies should be further investigated to draw metabolomic pathway associations with PCa progression and identify mechanisms through which metabolomic pathways respond to RT and other treatments. This study is also limited by the lack of validation of the pathways associated with progression and metastasis that could lead to the integration of identified altered pathways into a risk assessment profile for predictive purposes. These limitations could be addressed by the future availability of funding and access to larger PCa databases.

In conclusion and for future directions, this precision phylometabolomic analysis could be used to assess metabolomic pathways altered in other types of cancer and for response to other treatments. The determination of individual metabolic profiles and associated clinical phenotypes may allow precision phylometabolomics to provide clinically relevant personalized treatment and targeted therapeutic plans for a range of cancers and treatments, beginning with PCa and RT.

## Figures and Tables

**Table 1:** Demographics, clinical profile, and treatment plan of study participants.

		No of Patients (N = 55)	(%)
Demographics			
Age	Median 68 (52–90)		
Race	Black	15	27.2
	White	37	67.2
	Other	3	5.6
Clinical and Pathological Characteristics			
Pretreatment PSA (ng/mL)	Median 8.1 (1.9–25.6)		
T-stage	T1c	40	72.7
	T2a-b	12	21.8
	T2c	3	5.5
Gleason Score	6 (3 + 3)	20	36.3
	7(3 + 4; 4 + 3)	30	54.5
	8(3 + 5; 4 + 4)	4	7.2
	9(4 + 5; 5 + 4)	1	2
D’Amico classification			
Risk Group (D’Amico classification)	Low	14	25.4
	Intermediate	34	61.8
	High	7	12.8
Treatment Plan			
ADT	Yes	13	23.6
	No	42	76.4
Treatment: SBRT (CK) only	Fraction 5; Dose (Gy) 35	6	10.9
	Fraction 5; Dose (Gy) 36.25	36	65.4
Treatment: SBRT/IMRT combination	Fraction 3/25; Dose (Gy) 19.5/45	9	16.3
	Fraction 3/28; Dose (Gy) 19.5/50.4	4	7.2

**Table 2:** Biosignatures of study participants.

Risk Assessment	Nº of Patients	Treatment Protocol	Precision Biosignatures
High	7	Pre-RT	Hypoxanthine m/z 159.0093618 m/z 120.0038 m/z 380.772 m/z 197.08
		Post-RT	Phthalic acid 5′-Benzoylphosphoadenosine Bilirubin Phosphatidylethanolamine Phosphatidylcholine m/z 416.91035669 m/z 632.2994 m/z 312.0288 m/z 262.2532 m/z 184.0735 m/z 723.5443 m/z 701.561
Metastatic [2 high risk; 6 intermediate; 3 low risk]	11	Pre-RT	D-Tryptophan Hypoxanthine Tetrahydroisoquinoline Dihydrosanguinarine Methylglutaric acid
		Post-RT	Carbamic acid Phosphoric acid Bilirubin Phthalic acid 5′-Benzoylphosphoadenosine.

## Data Availability

The dataset is available upon request from the authors. No repository was created for the datasets, and the algorithm used was patented (US patent #US8849576B2). A detailed description of the analytical tool and its application was published in 2015 [35].

## Abbreviations

The following abbreviations are used in this manuscript:

ADT	Androgen deprivation therapy
PCa	Prostate cancer
PE	Phosphatidylethanolamine
PC	Phosphatidylcholine
RT	Radiotherapy (radiation treatment)
SBRT	Stereotactic body radiation therapy

## References

[1] Jemal A., Bray F., Center M.M., Ferlay J., Ward E., Forman D. (2011). Global Cancer Statistics. CA Cancer J. Clin..

[2] Ferlay J., Soerjomataram I., Dikshit R., Eser S., Mathers C., Rebelo M., Parkin D.M., Forman D., Bray F. (2015). Cancer Incidence and Mortality Worldwide: Sources, Methods and Major Patterns in GLOBOCAN 2012. Int. J. Cancer.

[3] Rawla P. (2019). Epidemiology of Prostate Cancer. World J. Oncol..

[4] Schafer E.J., Laversanne M., Sung H., Soerjomataram I., Briganti A., Dahut W., Bray F., Jemal A. (2025). Recent Patterns and Trends in Global Prostate Cancer Incidence and Mortality: An Update. Eur. Urol..

[5] Medipally D.K.R., Nguyen T.N.Q., Bryant J., Untereiner V., Sockalingum G.D., Cullen D., Noone E., Bradshaw S., Finn M., Dunne M. (2019). Monitoring Radiotherapeutic Response in Prostate Cancer Patients Using High Throughput FTIR Spectroscopy of Liquid Biopsies. Cancers.

[6] Bolla M., Gonzalez D., Warde P., Dubois J.B., Mirimanoff R.O., Storme G., Bernier J., Kuten A., Sternberg C., Gil T. (1997). Improved Survival in Patients with Locally Advanced Prostate Cancer Treated with Radiotherapy and Goserelin. N. Engl. J. Med..

[7] Jones C.U., Hunt D., McGowan D.G., Amin M.B., Chetner M.P., Bruner D.W., Leibenhaut M.H., Husain S.M., Rotman M., Souhami L. (2011). Radiotherapy and Short-Term Androgen Deprivation for Localized Prostate Cancer. N. Engl. J. Med..

[8] Shipley W.U., Seiferheld W., Lukka H.R., Major P.P., Heney N.M., Grignon D.J., Sartor O., Patel M.P., Bahary J.-P., Zietman A.L. (2017). Radiation with or without Antiandrogen Therapy in Recurrent Prostate Cancer. N. Engl. J. Med..

[9] Crawford E.D., Heidenreich A., Lawrentschuk N., Tombal B., Pompeo A.C.L., Mendoza-Valdes A., Miller K., Debruyne F.M.J., Klotz L. (2019). Androgen-Targeted Therapy in Men with Prostate Cancer: Evolving Practice and Future Considerations. Prostate Cancer Prostatic Dis..

[10] Tarish F.L., Schultz N., Tanoglidi A., Hamberg H., Letocha H., Karaszi K., Hamdy F.C., Granfors T., Helleday T. (2015). Castration Radiosensitizes Prostate Cancer Tissue by Impairing DNA Double-Strand Break Repair. Sci. Transl. Med..

[11] Sorrentino C., Musiani P., Pompa P., Cipollone G., Di Carlo E. (2011). Androgen Deprivation Boosts Prostatic Infiltration of Cytotoxic and Regulatory T Lymphocytes and Has No Effect on Disease-Free Survival in Prostate Cancer Patients. Clin. Cancer Res..

[12] Escamilla J., Schokrpur S., Liu C., Priceman S.J., Moughon D., Jiang Z., Pouliot F., Magyar C., Sung J.L., Xu J. (2015). CSF1 Receptor Targeting in Prostate Cancer Reverses Macrophage-Mediated Resistance to Androgen Blockade Therapy. Cancer Res..

[13] Hsueh J.Y., Gallagher L., Koh M.J., Shah S., Danner M., Zwart A., Ayoob M., Kumar D., Leger P., Dawson N.A. (2024). The Impact of Neoadjuvant Relugolix on Multi-Dimensional Patient-Reported Fatigue. Front. Oncol..

[14] Thompson I.M., Pauler D.K., Goodman P.J., Tangen C.M., Lucia M.S., Parnes H.L., Minasian L.M., Ford L.G., Lippman S.M., Crawford E.D. (2004). Prevalence of Prostate Cancer among Men with a Prostate-Specific Antigen Level < or =4.0 Ng per Milliliter. N. Engl. J. Med..

[15] Saini S. (2016). PSA and beyond: Alternative Prostate Cancer Biomarkers. Cell. Oncol..

[16] Giskeødegård G.F., Madssen T.S., Euceda L.R., Tessem M.-B., Moestue S.A., Bathen T.F. (2019). NMR-based metabolomics of biofluids in cancer. NMR Biomed..

[17] Alpert P.F. (2018). New Evidence for the Benefit of Prostate-Specific Antigen Screening: Data From 400,887 Kaiser Permanente Patients. Urology.

[18] Tutrone R., Lowentritt B., Neuman B., Donovan M.J., Hallmark E., Cole T.J., Yao Y., Biesecker C., Kumar S., Verma V. (2023). ExoDx Prostate Test as a Predictor of Outcomes of High-Grade Prostate Cancer—An Interim Analysis. Prostate Cancer Prostatic Dis..

[19] Visser W.C.H., de Jong H., Steyaert S., Melchers W.J.G., Mulders P.F.A., Schalken J.A. (2022). Clinical Use of the mRNA Urinary Biomarker SelectMDx Test for Prostate Cancer. Prostate Cancer Prostatic Dis..

[20] Michigan Center for Translational Pathology. https://mlabs.umich.edu/tests/myprostatescoretm-mps.

[21] Becerra M.F., Bhat A., Mouzannar A., Atluri V.S., Punnen S. (2019). Serum and Urinary Biomarkers for Detection and Active Surveillance of Prostate Cancer. Curr. Opin. Urol..

[22] Wang K., Wang X., Pan Q., Zhao B. (2023). Liquid Biopsy Techniques and Pancreatic Cancer: Diagnosis, Monitoring, and Evaluation. Mol. Cancer.

[23] Blanchard P., Briganti A., Bossi A. (2016). Re: Christopher J.D. Wallis, Refik Saskin, Richard Choo; et al. Surgery Versus Radiotherapy for Clinically-Localized Prostate Cancer: A Systematic Review and Meta-Analysis. Eur Urol 2016;70:21–30. Eur. Urol..

[24] Lokhov P.G., Dashtiev M.I., Bondartsov L.V., Lisitsa A.V., Moshkovskiĭ S.A., Archakov A.I. (2009). Metabolic fingerprinting of blood plasma for patients with prostate cancer. Biomed. Khim..

[25] Trock B.J. (2011). Application of Metabolomics to Prostate Cancer. Urol. Oncol..

[26] Fritz K.S., Petersen D.R. (2011). Exploring the Biology of Lipid Peroxidation Derived Protein Carbonylation. Chem. Res. Toxicol..

[27] Milosevic M., Warde P., Ménard C., Chung P., Toi A., Ishkanian A., McLean M., Pintilie M., Sykes J., Gospodarowicz M. (2012). Tumor Hypoxia Predicts Biochemical Failure Following Radiotherapy for Clinically Localized Prostate Cancer. Clin. Cancer Res..

[28] Moren X., Lhomme M., Bulla A., Sanchez J.-C., Kontush A., James R.W. (2016). Proteomic and Lipidomic Analyses of Paraoxonase Defined High Density Lipoprotein Particles: Association of Paraoxonase with the Anti-Coagulant, Protein S. Proteomics Clin. Appl..

[29] Hall W.A., Lawton C.A., Jani A.B., Pollack A., Feng F.Y. (2017). Biomarkers of Outcome in Patients with Localized Prostate Cancer Treated With Radiotherapy. Semin. Radiat. Oncol..

[30] Keam S.P., Caramia F., Gamell C., Paul P.J., Arnau G.M., Neeson P.J., Williams S.G., Haupt Y. (2018). The Transcriptional Landscape of Radiation-Treated Human Prostate Cancer: Analysis of a Prospective Tissue Cohort. Int. J. Radiat. Oncol. Biol. Phys..

[31] Chen C., Wang J., Pan D., Wang X., Xu Y., Yan J., Wang L., Yang X., Yang M., Liu G. (2023). Applications of Multi-omics Analysis in Human Diseases. MedComm.

[32] Gowda G.A.N., Zhang S., Gu H., Asiago V., Shanaiah N., Raftery D. (2008). Metabolomics-Based Methods for Early Disease Diagnostics. Expert Rev. Mol. Diagn..

[33] Vander Heiden M.G. (2011). Targeting Cancer Metabolism: A Therapeutic Window Opens. Nat. Rev. Drug Discov..

[34] Yu L., Li K., Zhang X. (2017). Next-Generation Metabolomics in Lung Cancer Diagnosis, Treatment and Precision Medicine: Mini Review. Oncotarget.

[35] Salazar J., Amri H., Noursi D., Abu-Asab M. (2015). Computational Tools for Parsimony Phylogenetic Analysis of Omics Data. OMICS.

[36] Somarelli J.A., Ware K.E., Kostadinov R., Robinson J.M., Amri H., Abu-Asab M., Fourie N., Diogo R., Swofford D., Townsend J.P. (2017). PhyloOncology: Understanding Cancer through Phylogenetic Analysis. Biochim. Biophys. Acta Rev. Cancer.

[37] Nalbantoglu S., Abu-Asab M., Suy S., Collins S., Amri H. (2019). Metabolomics-Based Biosignatures of Prostate Cancer in Patients Following Radiotherapy. OMICS.

[38] Abu-Asab M.S., Chaouchi M., Amri H. (2008). Phylogenetic Modeling of Heterogeneous Gene-Expression Microarray Data from Cancerous Specimens. OMICS.

[39] Nalbantoglu S., Abu-Asab M., Tan M., Zhang X., Cai L., Amri H. (2016). Study of Clinical Survival and Gene Expression in a Sample of Pancreatic Ductal Adenocarcinoma by Parsimony Phylogenetic Analysis. OMICS.

[40] Azzone G., Manzini R., Noci G. (1996). Evolutionary Trends in Environmental Reporting. Bus. Strategy Environ..

[41] (2011). Phylogenetic Classification. Phylogenetics.

[42] Xiao J.F., Varghese R.S., Zhou B., Nezami Ranjbar M.R., Zhao Y., Tsai T.-H., Di Poto C., Wang J., Goerlitz D., Luo Y. (2012). LC–MS Based Serum Metabolomics for Identification of Hepatocellular Carcinoma Biomarkers in Egyptian Cohort. J. Proteome Res..

[43] Smith C.A., Want E.J., O’Maille G., Abagyan R., Siuzdak G. (2006). XCMS: Processing Mass Spectrometry Data for Metabolite Profiling Using Nonlinear Peak Alignment, Matching, and Identification. Anal. Chem..

[44] Abu-Asab M., Chaouchi M., Amri H. (2006). Phyloproteomics: What Phylogenetic Analysis Reveals about Serum Proteomics. J. Proteome Res..

[45] Felsenstein J. (1989). PHYLIP-Phylogeny Inference Package (Version 3.2). Cladistics.

[46] Page R.D. (1996). TreeView: An Application to Display Phylogenetic Trees on Personal Computers. Comput. Appl. Biosci..

[47] Wishart D.S., Jewison T., Guo A.C., Wilson M., Knox C., Liu Y., Djoumbou Y., Mandal R., Aziat F., Dong E. (2013). HMDB 3.0—The Human Metabolome Database in 2013. Nucleic Acids Res..

[48] Cui Q., Lewis I.A., Hegeman A.D., Anderson M.E., Li J., Schulte C.F., Westler W.M., Eghbalnia H.R., Sussman M.R., Markley J.L. (2008). Metabolite Identification via the Madison Metabolomics Consortium Database. Nat. Biotechnol..

[49] Tautenhahn R., Cho K., Uritboonthai W., Zhu Z., Patti G.J., Siuzdak G. (2012). An Accelerated Workflow for Untargeted Metabolomics Using the METLIN Database. Nat. Biotechnol..

[50] Sud M., Fahy E., Cotter D., Brown A., Dennis E.A., Glass C.K., Merrill A.H., Murphy R.C., Raetz C.R.H., Russell D.W. (2007). LMSD: LIPID MAPS Structure Database. Nucleic Acids Res..

[51] Kanehisa M., Furumichi M., Tanabe M., Sato Y., Morishima K. (2017). KEGG: New Perspectives on Genomes, Pathways, Diseases and Drugs. Nucleic Acids Res..

[52] Kamburov A., Stelzl U., Lehrach H., Herwig R. (2013). The ConsensusPathDB Interaction Database: 2013 Update. Nucleic Acids Res..

[53] Paydar I., Cyr R.A., Yung T.M., Lei S., Collins B.T., Chen L.N., Suy S., Dritschilo A., Lynch J.H., Collins S.P. (2016). Proctitis 1 Week after Stereotactic Body Radiation Therapy for Prostate Cancer: Implications for Clinical Trial Design. Front. Oncol..

[54] Danner M., Hung M.-Y., Yung T.M., Ayoob M., Lei S., Collins B.T., Suy S., Collins S.P. (2017). Utilization of Patient-Reported Outcomes to Guide Symptom Management during Stereotactic Body Radiation Therapy for Clinically Localized Prostate Cancer. Front. Oncol..

[55] Mercado C., Kress M.-A., Cyr R.A., Chen L.N., Yung T.M., Bullock E.G., Lei S., Collins B.T., Satinsky A.N., Harter K.W. (2016). Intensity-Modulated Radiation Therapy with Stereotactic Body Radiation Therapy Boost for Unfavorable Prostate Cancer: The Georgetown University Experience. Front. Oncol..

[56] D’Amico A.V., Whittington R., Malkowicz S.B., Schultz D., Blank K., Broderick G.A., Tomaszewski J.E., Renshaw A.A., Kaplan I., Beard C.J. (1998). Biochemical Outcome after Radical Prostatectomy, External Beam Radiation Therapy, or Interstitial Radiation Therapy for Clinically Localized Prostate Cancer. JAMA.

[57] Edison A.S., Hall R.D., Junot C., Karp P.D., Kurland I.J., Mistrik R., Reed L.K., Saito K., Salek R.M., Steinbeck C. (2016). The Time Is Right to Focus on Model Organism Metabolomes. Metabolites.

[58] Ma S., Yim S.H., Lee S.-G., Kim E.B., Lee S.-R., Chang K.-T., Buffenstein R., Lewis K.N., Park T.J., Miller R.A. (2015). Organization of the Mammalian Metabolome According to Organ Function, Lineage Specialization, and Longevity. Cell Metab..

[59] Loeb K.R., Loeb L.A. (2000). Significance of Multiple Mutations in Cancer. Carcinogenesis.

[60] Chung D.C. (2000). The Genetic Basis of Colorectal Cancer: Insights into Critical Pathways of Tumorigenesis. Gastroenterology.

[61] Hayashi Y., Yamashita J., Watanabe T. (2004). Molecular Genetic Analysis of Deep-Seated Glioblastomas. Cancer Genet. Cytogenet..

[62] Adsay N.V., Merati K., Andea A., Sarkar F., Hruban R.H., Wilentz R.E., Goggins M., Iocobuzio-Donahue C., Longnecker D.S., Klimstra D.S. (2002). The Dichotomy in the Preinvasive Neoplasia to Invasive Carcinoma Sequence in the Pancreas: Differential Expression of MUC1 and MUC2 Supports the Existence of Two Separate Pathways of Carcinogenesis. Mod. Pathol..

[63] Petricoin E.E., Paweletz C.P., Liotta L.A. (2002). Clinical Applications of Proteomics: Proteomic Pattern Diagnostics. J. Mammary Gland Biol. Neoplasia.

[64] Alexe G., Alexe S., Liotta L.A., Petricoin E., Reiss M., Hammer P.L. (2004). Ovarian Cancer Detection by Logical Analysis of Proteomic Data. Proteomics.

[65] Conrads T.P., Fusaro V.A., Ross S., Johann D., Rajapakse V., Hitt B.A., Steinberg S.M., Kohn E.C., Fishman D.A., Whitely G. (2004). High-Resolution Serum Proteomic Features for Ovarian Cancer Detection. Endocr. Relat. Cancer.

[66] Zhu W., Wang X., Ma Y., Rao M., Glimm J., Kovach J.S. (2003). Detection of Cancer-Specific Markers amid Massive Mass Spectral Data. Proc. Natl. Acad. Sci. USA.

[67] Adam B.-L., Qu Y., Davis J.W., Ward M.D., Clements M.A., Cazares L.H., Semmes O.J., Schellhammer P.F., Yasui Y., Feng Z. (2002). Serum Protein Fingerprinting Coupled with a Pattern-Matching Algorithm Distinguishes Prostate Cancer from Benign Prostate Hyperplasia and Healthy Men. Cancer Res..

[68] Tan J.S.H., Lin X., Chua K.L.M., Lam P.Y., Soo K.-C., Chua M.L.K. (2017). Exploiting Molecular Genomics in Precision Radiation Oncology: A Marriage of Biological and Physical Precision. Chin. Clin. Oncol..

[69] Laiakis E.C., Mak T.D., Anizan S., Amundson S.A., Barker C.A., Wolden S.L., Brenner D.J., Fornace A.J. (2014). Development of a Metabolomic Radiation Signature in Urine from Patients Undergoing Total Body Irradiation. Radiat. Res..

[70] Lacombe J., Mange A., Azria D., Solassol J. (2013). Identification of predictive biomarkers to radiotherapy outcome through proteomics approaches. Cancer Radiother..

[71] Coates J., Jeyaseelan A.K., Ybarra N., David M., Faria S., Souhami L., Cury F., Duclos M., El Naqa I. (2015). Contrasting Analytical and Data-Driven Frameworks for Radiogenomic Modeling of Normal Tissue Toxicities in Prostate Cancer. Radiother. Oncol..

[72] Christensen E., Evans K.R., Ménard C., Pintilie M., Bristow R.G. (2008). Practical Approaches to Proteomic Biomarkers within Prostate Cancer Radiotherapy Trials. Cancer Metastasis Rev..

[73] Abu-Asab M., Zhang M., Amini D., Abu-Asab N., Amri H. (2011). Endometriosis Gene Expression Heterogeneity and Biosignature: A Phylogenetic Analysis. Obstet. Gynecol. Int..

[74] Abu-Asab M., Chaouchi M., Amri H. (2008). Evolutionary Medicine: A Meaningful Connection between Omics, Disease, and Treatment. Proteomics Clin. Appl..

[75] Sarnat H.B., Netsky M.G. (1984). Hypothesis: Phylogenetic Diseases of the Nervous System. Can. J. Neurol. Sci..

[76] Putluri N., Shojaie A., Vasu V.T., Nalluri S., Vareed S.K., Putluri V., Vivekanandan-Giri A., Byun J., Pennathur S., Sana T.R. (2011). Metabolomic Profiling Reveals a Role for Androgen in Activating Amino Acid Metabolism and Methylation in Prostate Cancer Cells. PLoS ONE.

[77] Sreekumar A., Poisson L.M., Rajendiran T.M., Khan A.P., Cao Q., Yu J., Laxman B., Mehra R., Lonigro R.J., Li Y. (2009). Metabolomic Profiles Delineate Potential Role for Sarcosine in Prostate Cancer Progression. Nature.

[78] Nakajima T., Vares G., Wang B., Nenoi M. (2016). Chronic Intake of Japanese Sake Mediates Radiation-Induced Metabolic Alterations in Mouse Liver. PLoS ONE.

[79] Yin J., Ren W., Huang X., Deng J., Li T., Yin Y. (2018). Potential Mechanisms Connecting Purine Metabolism and Cancer Therapy. Front. Immunol..

[80] Pannkuk E.L., Laiakis E.C., Mak T.D., Astarita G., Authier S., Wong K., Fornace A.J. (2016). A Lipidomic and Metabolomic Serum Signature from Nonhuman Primates Exposed to Ionizing Radiation. Metabolomics.

[81] Guo C., Wu L., Xu L. (2025). Mendelian Randomization Analysis Reveals Potential Causal Relationships between Serum Lipid Metabolites and Prostate Cancer Risk. Discov. Oncol..

[82] Jelonek K., Pietrowska M., Ros M., Zagdanski A., Suchwalko A., Polanska J., Marczyk M., Rutkowski T., Skladowski K., Clench M.R. (2014). Radiation-Induced Changes in Serum Lipidome of Head and Neck Cancer Patients. Int. J. Mol. Sci..

[83] Kurmi K., Haigis M.C. (2020). Nitrogen Metabolism in Cancer and Immunity. Trends Cell Biol..

[84] Kodama M., Oshikawa K., Shimizu H., Yoshioka S., Takahashi M., Izumi Y., Bamba T., Tateishi C., Tomonaga T., Matsumoto M. (2020). A Shift in Glutamine Nitrogen Metabolism Contributes to the Malignant Progression of Cancer. Nat. Commun..

[85] Maitra D., Bragazzi Cunha J., Elenbaas J.S., Bonkovsky H.L., Shavit J.A., Omary M.B. (2019). Porphyrin-Induced Protein Oxidation and Aggregation as a Mechanism of Porphyria-Associated Cell Injury. Cell. Mol. Gastroenterol. Hepatol..

[86] Zhang H., Bo S., Zeng K., Wang J., Li Y., Yang Z., Zhou X., Chen S., Jiang Z.-X. (2020). Fluorinated Porphyrin-Based Theranostics for Dual Imaging and Chemo-Photodynamic Therapy. J. Mater. Chem. B.

[87] Boss M.-K., Oberley-Deegan R.E., Batinic-Haberle I., Talmon G.A., Somarelli J.A., Xu S., Kosmacek E.A., Griess B., Mir S., Shrishrimal S. (2021). Manganese Porphyrin and Radiotherapy Improves Local Tumor Response and Overall Survival in Orthotopic Murine Mammary Carcinoma Models. Radiat. Res..

[88] Kim M., Tomek P. (2021). Tryptophan: A Rheostat of Cancer Immune Escape Mediated by Immunosuppressive Enzymes IDO1 and TDO. Front. Immunol..

[89] Della Chiesa M., Carlomagno S., Frumento G., Balsamo M., Cantoni C., Conte R., Moretta L., Moretta A., Vitale M. (2006). The Tryptophan Catabolite L-Kynurenine Inhibits the Surface Expression of NKp46- and NKG2D-Activating Receptors and Regulates NK-Cell Function. Blood.

[90] Rad Pour S., Morikawa H., Kiani N.A., Yang M., Azimi A., Shafi G., Shang M., Baumgartner R., Ketelhuth D.F.J., Kamleh M.A. (2019). Exhaustion of CD4+ T-Cells Mediated by the Kynurenine Pathway in Melanoma. Sci. Rep..

[91] Mezrich J.D., Fechner J.H., Zhang X., Johnson B.P., Burlingham W.J., Bradfield C.A. (2010). An Interaction between Kynurenine and the Aryl Hydrocarbon Receptor Can Generate Regulatory T Cells. J. Immunol..

[92] Liu Y., Liang X., Dong W., Fang Y., Lv J., Zhang T., Fiskesund R., Xie J., Liu J., Yin X. (2018). Tumor-Repopulating Cells Induce PD-1 Expression in CD8+ T Cells by Transferring Kynurenine and AhR Activation. Cancer Cell.

[93] Shao Y., Zhang X., Zhang Y., Liu Z., Yang Z., Liu Y., Huang H., Wang Z., Fu Z., Wang Y. (2025). Development and Validation of Tryptophan Metabolism-Related Risk Model and Molecular Subtypes for Predicting Postoperative Biochemical Recurrence in Prostate Cancer. Transl. Androl. Urol..

[94] Inoguchi T., Nohara Y., Nojiri C., Nakashima N. (2021). Association of Serum Bilirubin Levels with Risk of Cancer Development and Total Death. Sci. Rep..

[95] Glunde K., Bhujwalla Z.M., Ronen S.M. (2011). Choline Metabolism in Malignant Transformation. Nat. Rev. Cancer.

[96] Giskeødegård G.F., Hansen A.F., Bertilsson H., Gonzalez S.V., Kristiansen K.A., Bruheim P., Mjøs S.A., Angelsen A., Bathen T.F., Tessem M.-B. (2015). Metabolic Markers in Blood Can Separate Prostate Cancer from Benign Prostatic Hyperplasia. Br. J. Cancer.

[97] Yoo B.C., Kong S.-Y., Jang S.-G., Kim K.-H., Ahn S.-A., Park W.-S., Park S., Yun T., Eom H.-S. (2010). Identification of Hypoxanthine as a Urine Marker for Non-Hodgkin Lymphoma by Low-Mass-Ion Profiling. BMC Cancer.

[98] Long Y., Sanchez-Espiridion B., Lin M., White L., Mishra L., Raju G.S., Kopetz S., Eng C., Hildebrandt M.A.T., Chang D.W. (2017). Global and Targeted Serum Metabolic Profiling of Colorectal Cancer Progression. Cancer.

[99] Russo P., Bizzarri F.P., Filomena G.B., Marino F., Iacovelli R., Ciccarese C., Boccuto L., Ragonese M., Gavi F., Rossi F. (2024). Relationship Between Loss of Y Chromosome and Urologic Cancers: New Future Perspectives. Cancers.

